# Nurses Practice Beyond Simple Advocacy to Engage in Relational Narratives: Expanding Opportunities for Persons to Influence the Public Space

**DOI:** 10.2174/1874434600802010040

**Published:** 2008-05-13

**Authors:** N Murphy, C Aquino-Russell

**Affiliations:** 1 Dalhousie University School of Nursing, 5869 University Avenue, Halifax Nova Scotia, B3H 4P5, Canada; 2 University of New Brunswick School of Nursing, Moncton Campus, Moncton, New Brunswick, E1C 4B7, Canada

**Keywords:** Health inequalities, existential advocacy, relational narrative, respect, discernment of values, character formation.

## Abstract

In practicing existential and human advocacy, or engaging in a relational narrative, nurses may assist persons who experience health inequalities to clarify their values, and, in becoming more fully their authentic selves, community members who ordinarily feel powerless in the public space may act with confidence in influencing the distribution of health-care resources. In this paper, the writers describe research characterizing nurses’ advocacy practices and review the concepts of respect and self-interpretation as a foundation for arguing that nurses who engage in relational narratives with the persons they serve may encourage continuing acts of self-understanding. Investigators indicated that nurses characterized their practices as a therapeutic endeavor, and that their practices were grounded in respect. Practicing nurses may need self-awareness to habitually convey respect for human dignity, in addition, nurse educators ought to attend to the professional development of student nurses, providing opportunities for the formation of character traits or qualities.

## INTRODUCTION

Many nurses are mindful that persons who experience health inequalities related to socio-economic and political circumstances have a right to contribute to public policy-making that affects the distribution of resources, including health care. Concepts of deliberative democracy have drawn our attention to the ideal that all persons have a right to contribute to public decisions that affect their lives [1]. Nevertheless, many community members who are less advantaged socially often feel powerless to influence public policy-making [2], and further, many nurses have yet to develop a practice whereby they assist community members to speak on their own behalf to policy-makers. Grace [3] urged nurses to widen their scope of accountability to include professional advocacy that “commits (nurses) to a more holistic view of health, persons and environments”. Although nurses have a tradition of advocating for clients within their practice settings, the notion of professional advocacy advanced by Grace calls nurses to establish relationships with the persons they serve that not only encourage these persons to represent their needs to policy-makers, but may ultimately influence a just distribution of resources that mitigate harmful social conditions.

The theory of advocacy has evolved in the past two decades such that conceptual contexts are available for nurses to think about avenues for assisting persons who seek to take a stand in the public space. By custom, nurses practice simple or consumer-centric advocacy by representing clients’ needs, as well as by teaching clients about their health-care options [4, 5]. Reaching beyond these forms of advocacy to support persons who want to speak on their own behalf, nurses may employ concepts of existential advocacy [6] and human advocacy [7]; Gadow and Curtin articulated how nurses may assist persons to discern their values, or gain self-understanding, and thus act grounded in explicitly-known values [8]. More precisely, Gadow [9] contended that through inter-subjectivity, or, engagement in a relational narrative, nurses may be present to persons as they clarify their values, and thus transform experiences of disease and suffering into experiences of personal development. A relational narrative may be defined as inter-subjectivity between a nurse and a person such that, in a nurse’s presence, the person not only expands his or her consciousness of deeply-held values, but also acts to express those values, thus becoming his or her authentic self. Gadow stressed, moreover, that gaining a discerning insight into one’s values entails continuing acts of interpretation. Furthermore, Curtin asserted that due to persons sharing a common humanity, nurses ought to share their self-knowledge with persons as they search for meaning in their experiences.

The investigators of qualitative research studies undertaken over the past decade to understand nurses’ characterization of their advocacy practices suggested that some nurses may be practicing beyond simple and consumer-centric advocacy to engage in relational narratives with the persons they serve. Snowball [10], for example, concluded that nurse participants conceptualized their advocacy practices as a “therapeutic endeavor”. Furthermore, Watt’s [11] participants identified respect for inherent human characteristics, including the ability to make choices that are congruent with needs, as fundamental to their nursing practices. Through their study, Vaartio, Leino-Kilpi, Salantera and Suominen [12] reinforced these ideas of practice, specifically, that interpersonal dialogue, respect and empowerment are robust components of advocacy. Similarly, in a qualitative study of the universal lived experience of feeling respected, Parse’s [13] study participants reported that “feeling respected happens… with being validated by others”. Further, participants stressed that recognition of one’s competence is often the source of validation; as well, recognition of the person one is becoming is important in thinking about the quality of one’s life. Atkins [14] reinforced this notion by stressing that persons’ competence in making autonomous choices is influenced by nurses’ relational approaches, such as empathy that in turn leads to persons’ insight into values and beliefs, and ultimately self-expression.

Through the aforementioned studies, researchers suggested that respect is pivotal to nurse-person relatedness that encourages persons in their self-development. Recall, Parse [13] suggested that feeling respected is connected to feeling competent in becoming who one aspires to be. Yet an articulation of the concept of respect is needed to better understand why nurses’ conveyance of respect matters to persons as they struggle to understand their experiences. To clarify this idea, Dillon [15] asserted that who we become as persons depends significantly on how we are perceived and treated by others. To summarize her point, Dillon stressed that we have “power to make and unmake each other as persons,” and therefore respect for others requires that one tries to understand what it is for the other to live the life he or she lives [16]. In conveying respect, nurses may encourage persons to continue acts of interpretation, and thus discern their values and choose actions that express these values.

To further delineate the argument of nurse-person relatedness, the writers of this paper will: (a) outline elements of Gadow’s [6] existential advocacy, as well as Curtin’s [7] human advocacy; (b) discuss research evidence related to advocacy practices of nurses that indicated some nurses may engage in a relational narrative as an avenue to assist persons in the discernment of their values, including findings by Snowball [10], Watt [11], Vaartio, Leino-Kilpi, Salantera and Suominen [12] Foley, Minick and Kee [17] and Chafey, Rhea & Spencer [18]. Moreover, the writers will: (c) explore the meaning of the concept of respect for self and others [15, 19, 20]; (d) examine how nurses may convey respect that encourages persons to undertake continuing acts of interpretation by which they may discern their values and choose actions that express those values [8, 21, 22]; and (e) discuss implications for nursing practice, specifically, awareness and exercise of the value of respect, as well as implications for nursing education.

Through self-awareness and the exercise of respect, nurses may assist persons who experience health inequalities, and who may feel powerless, to become more fully their authentic selves, and, with a strong self-understanding, influence confidently the distribution of public resources that mitigate harmful socio-economic and political circumstances. Williams, Labonte and O’Brien [23] concluded that marginalized community members who consciously articulate their personal identities prior to political activism may be more effective in their efforts.

## THE RELATIONAL NARRATIVE AND PERSONS’ DISCERNMENT OF VALUES

Referred to as existential advocacy by Gadow in the 1980s, engagement in a relational narrative [9] is integral to nursing practice due to its relevance to persons’ self-development [24]. In this discussion, the idea of engagement in a relational narrative will thus be applied, as well as the notion that self-knowledge is shaped by particular sets of values and contexts, including events and relationships. The tenets of existential advocacy and engagement in a relational narrative are consistent, as Gadow [6] demonstrated by cautioning that existential advocacy is distinct from paternalism and consumer protection, due to its basis in the principle of freedom of self-determination. Nonetheless, advocacy is a legal term denoting activism or speaking on behalf of powerless individuals, hence the ambiguity of the connection between existential advocacy and engagement in a relational narrative, with the latter requiring the person’s capacity to act on his or her own behalf. Nurse-person engagement in a relational narrative is a more accessible term and will thus be used in this paper. Specifically, Gadow [6, 9] argued that through engagement in a relational narrative — that is premised in the belief that both the nurse and the person are moving toward their good — a person gains a discerning insight into the unique meaning of his or her experience, and thus can act to bring about coherence in a life experience.

To illuminate, Gadow [9] stipulated that the most reliable way for a person to know the good he or she seeks is through his or her own accounts, and thus posited that the relational narrative is a means for persons’ discernment of values or ethical knowing that is particular and contextual. Gadow believed that disease and suffering offers persons an opportunity to self-reflect, or, stated differently, a time to make choices about the kind of person one wants to become. She argued that disease may “overturn” [6] emotions and spiritual values, generating a feeling of “thing-like other[ness]” [6], or estrangement. In discerning or “reaffirming” [6] personal values, the experience of otherness can be transformed positively. Gadow simultaneously asserted that the nurse and person co-author the assignment of meaning, arguing that nurses’ respect for persons’ unique experiences is pivotal to the discernment process, as well, a discerning insight evolves within the context of particular values.

Similar to Gadow [6], Curtin [7] emphasized nurse-person relatedness by urging nurses to embrace concepts of humanism to facilitate persons in discovering purpose in their experiences. She believed that persons share a common humanity, stating, “We have grown out of the growth of others, learned from their knowledge and benefited from their suffering” [7]. Premised on this notion, Curtin explained that nurses should share insights with their clients that they have gained through their suffering in order to assist these persons in the interpretation of their suffering. Arman [25] extended Curtin’s argument by asserting unselfish actions by others enable a person to experience strength and confidence in his or her being.

Through engagement in relational narratives, nurses may facilitate persons in gaining an insight into their accounts of their good, and thus bring coherence into their experiences. Further, in conveying respect, nurses may encourage persons to continue the self-interpretation that is needed to discern deeply-held values.

## RESEARCH ADDRESSING NURSES’ PRACTICES

Researchers who have undertaken studies (Table **1**) to explore and describe nurses’ characterization of their advocacy practices supported the claim that nurses’ endeavors may be moving toward engagement in relational narratives, and more specifically, conveying respect for persons’ unique interpretations of their experiences. These investigators, including Snowball [10], Watt [11], Vaartio, Leino-Kilpi, Salantera and Suominen [12], Foley, Minick and Kee [17] and Chafey, Rhea & Spencer [18], demonstrated that nurses are aware of the essence of inter-subjective relatedness. Although Foley Minick and Kee [17] reported that nurses’ practices embrace earlier ideas of advocacy — including “safeguarding” persons’ integrity — overall, the findings revealed that some nurses practice according to the tenets of existentialism and humanism. The participants of the Foley, Minick and Kee study, for example, stressed that nurses must “engage in and understand the... dilemmas of the [person’s] life as [they are] actually lived”.

Moreover, Snowball’s [10] participants asserted that their advocacy was predicated on a therapeutic relationship, while seven of the same 15 participants stressed that nurses and persons share a “common humanity”. Snowball, however, did not provide the theoretical contexts used to organize the data patterns into categories, and, therefore, the constructs — therapeutic relationship and shared common humanity — are not theoretically known [26]. Nonetheless, one nurse participant from Snowball’s study described his or her practice as “imagining what it is like [for] the person experiencing an event, checking out his or her understanding of the experience, and working with the client’s response”. One may glean that this participant is respecting the person’s need to exercise his or her choice, or, to reframe in Gadow’s [6] terms, discern the meaning of his or her experience. Similar to Snowball’s participants, the Vaartio, Leino-Kilpi, Salantera and Suominen [12], Foley, Minick and Kee [17] and Chafey, Rhea and Spencer [18] study participants stipulated that interpersonal relatedness was central to their practices and Watt’s [11] participants reinforced this stance by asserting that respect was fundamental to advocacy.

Although nurses may be developing relational narratives with persons, research is needed to advance understanding of how nurses convey respect for persons’ ability to discern their values, and to choose actions that express those values. To understand more fully how nurses’ respect for persons’ knowledge of their values may shape the latter’s choices, an overview of the concept of respect will be provided.

## NURSES’ CONVEYANCE OF RESPECT FOR ALL PERSONS

In acting ethically, nurses are often preoccupied with ensuring clients have the information needed for making choices about treatment options overlooking at times that respect for autonomy entails regard for persons’ ability to reason and clarify their deeply-held values. To review the concept of respect may expand awareness of how nurses’ respect can encourage persons to continue their self-interpretation. Nurses have a duty to convey respect that preserves and promotes self-clarification, as identified by the Canadian Nurses Association Code of Ethics [32], which articulates primary values for nursing practice, including respect for the autonomy of all persons. Reinforcing this notion of practice, Roland and Foxx [20] stressed that we owe one another respect due to our status as moral beings, which means that we are able to conceptualize our own good [15] and to make choices that express that good. Taylor [33] summarized these thoughts in the assertion that respect serves to protect personal identity.

In understanding the need to re-assess the relationship between the concept of respect and persons’ capacity for reasoning about values, Roland and Foxx [20] discussed respect in terms of the dignity of self and others. Through providing a historical context of the concept of respect, they traced the notion of respect back to the writings of Aristotle, who posited that persons must act in a manner that respects the self. The 4^th^ century philosopher argued that a person must have an appreciation of his or her human dignity or worth and suggested further that moral actions are grounded in self-awareness, judgment and values. In a contemporary reflection on respect, Taylor [33] proposed that respecting the self entails living in accordance with personal standards and expectations that give one’s life its meaning. In advancing the discussion on this quality, Roland and Foxx recalled that Kant stressed that all persons deserve and are worthy of respect: “Act in such a way that you always treat humanity, whether in your own person or in the person of any other, never simply as a means, but always at the same time as an end” [20]. Furthermore, Roland and Foxx stated, that “Kant proposed that because of their ability to rationalize, think, and choose, individuals have a moral duty to respect others and themselves, which requires them to act in certain ways and not in others” [20].

In accordance with the Code of Ethics [32], nurses ought to practice in a manner that respects persons’ capacity to reason about their values and to examine actions that express those values, thus assisting them to transform experiences of suffering into experiences of personal development. Dillon [15] explicated further the nature of respect by asserting that self-respect is vulnerable to the perception and treatment of others, indicating that respect has a dialogic dimension. According to Cranor [19], a person is worthy of respect, a term derived from the latin word “respicere,” which means “to look back at,” because he or she is perceived as having “character-traits” or qualities that are regarded as worthwhile. In this case, the character trait that warrants respect is the capacity in both Aristotle’s perspective — to gain awareness of one’s values and to justify actions grounded in those values — and in Kant’s view — to reason and choose what gives value to one’s life. In respecting persons’ capacity to assign meaning in their lives, “one pays careful attention to” [15] the person’s feelings and thoughts in order to understand what it means to that person to live his or her life [16]. One may claim that trying to understand another’s lived experience is a Kantian moral duty — respect requires persons to act in certain ways and not in others. If persons have the capacity for reasoning about their values, whether in accordance with Aristotelian or Kantian thought, then their insights into the meaning of their lives are deserving of others’ respect. In feeling the respect of others, persons may conceptualize their own good, or who they aspire to be. Through her findings on feeling respected, Parse [13] indicated such potential, specifically, that feeling respected is feeling competent in one’s decision-making and validated in becoming one’s own person.

The significance of nurses’ conveyance of respect is well-recognized. Cody [34] reinforced the notion that nurses ought to be present to persons in their becoming. He asserted moreover, that nurses need to imagine what life is like for the other, seek to understand that life, and, in relating to the other, uphold the other’s human dignity unconditionally. Guided by ethical principles, including respect for human dignity, Cody urged nurses to attend to the feelings and thoughts of others as they assign meaning to their experiences. A theoretical context that depicts personal agency is needed to better understand this phenomenon of nurse-person relatedness.

## PERSONS AS SELF-INTERPRETING BEINGS

That persons plan their lives either by making objective, rational choices or by gaining an awareness of values embedded in their particular historical contexts, by which they judge the good life for the self, has been revealed. Charles Taylor [8, 21, 22], an eminent Canadian political theorist, asserted that persons are interpretative beings for whom things matter significantly. In his explication, Taylor contextualized further Gadow’s [6] argument that persons have the capacity to transform their experiences of disease and suffering into experiences of self-development through a discernment of values and an examination of actions that express those values. In addition, with his theory of personal agency, Taylor allows an exploration of the notion of feeling respected and self-knowledge, and thus aspects of his theory will follow.

As a person reasons about his or her values, he or she consciously becomes his or her authentic self. Taylor [8] asserted that persons seeks to identify themselves in a strong and original manner, simultaneously stressing that persons appraise their experiences in relation to standards that are embedded in their community practices, which are defined as “more or less stable configurations of shared activity” [22]. He contended as well that interpretations have emotional and cognitive components: feelings can be the points of access to the understanding of values [8]. Taylor [35] believed, moreover, that persons have a pre-understanding or dim awareness of the meaning of their predicaments, and thus how to respond. In examining the values underlying their feelings, what had been known dimly may be known explicitly. Beyond these premises, Taylor [8] asserted that in gaining a discerning insight into one’s values, a person may select one value to act upon rather than another, for example, to be other-regarding rather than self-regarding. Lastly, Taylor [8] stipulated that persons desire to assess whether they are meeting the standards they set for themselves, and, in this manner, persons realize their dignity as they articulate their pre-understanding and express particular values they identify as their own.

**Fig. (1):**
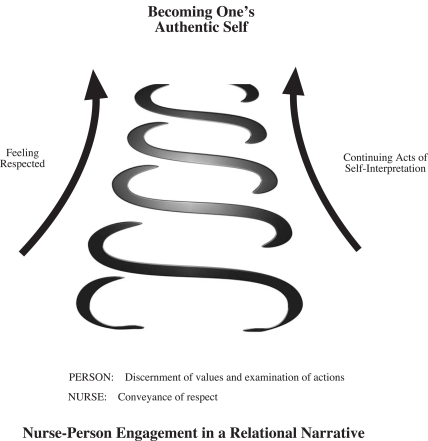
Depiction of nurse-person relatedness.

Continuing acts of self-interpretation often require not only personal courage, but also perseverance. Nonetheless, nurses’ conveyance of respect for personal dignity may encourage persons to continue in their self-knowledge in order to gain a discerning insight into the embedded values by which they deem life worth living (Fig. **1**). In feeling respected, persons can make rational transitions [35] in their self-understanding, for example, they may articulate a perception they had previously overlooked, and, in gaining awareness, respond to experiences, including suffering, in new and authentic ways.

## IMPLICATIONS FOR NURSING PRACTICE

Many nurses may desire to broaden their scope of accountability to include professional advocacy. Falk-Rafael [36] reinforced this ideal by discussing the nursing profession’s legacy of assisting persons who are socially disadvantaged, while Spenceley, Reutter and Allen [37] urged nurses to actively influence health-related public policy-making. To that end, nurses may engage in relational narratives with persons, who live with socio-economic and political conditions negatively associated with their health, and thus assist them to take a stand in the public space. In gaining confidence in their being, persons who had felt powerless may persuade health-related policy-makers to allocate resources to services and programs that mitigate harmful conditions.

Assisting persons to gain discerning insight into their experiences is challenging because self-awareness may be hindered by a lack of confidence. Gaining insight into embedded values can be emotionally and mentally rigorous, for example, persons may think they lack the capacity to achieve the goal. In addition, persons may misunderstand their experiences through irrationalities that replace their practical reasoning, as well they may take the viewpoints of others as their own [14]. Nurses who are willing to engage in relational narratives with persons who feel powerless in the public space may need to develop their self-awareness. The argument on self-awareness is warranted by several theorists, such as Vaartio, Leino-Kilpi, Salantera and Suominen [12], who asserted that nurses’ character and professional morality inform their practices, as well as Arman [25] who contended that caregivers have a responsibility to develop their moral being, and thus become closer to their authentic selves. Similarly, Atkins [14] argued that persons who seek to assist persons in their self-understanding need to be skilled in facilitating “critical self-reading”, and, more specifically she argued that nurses need insight into their character and motives, as well as a range of human ideas and emotions.

Character identity, or the habitual exercise of a trait, is a highly relevant concept for relational nursing practices due to the influence character identity has on perceptual judgment [38]. More precisely, a person’s disposition, or comportment, influences what he or she sees in a situation. Nurses who desire to engage in relational narratives, by which persons may gain insight into their authentic selves, ought to base their practices on an awareness and exercise of respect. The nurse’s perception that the person has the capacity to know what is the good for the self, even though his or her first or second interpretations may lack clarity, may encourage the person to continue his or her acts of self-interpretation grounded in particular community practices.

Acting with self-awareness, nurses who engage in relational narratives with community members who experience health inequalities may assist them to influence health policy-making. Moreover, nurses may enhance their effectiveness by applying Taylor’s [8, 21, 22, 35] theory of personal agency, which can function as scaffolding to structure their nurse-person relatedness, and thus community members’ personal development. As Taylor’s theory of self-interpretation is employed, community members may feel competent in representing their health interests to policy-makers owing, in part, to nurses’ recognition of their capacity for self-expression. More precisely, persons’ satisfaction of their need to strongly and originally identify their values, hence articulate who they want to become, may be a crucible in their capacity to persuade health policy-makers to respond to their needs. Such moments of self-clarification may be attained as persons appraise their feelings about everyday experiences in relation to their embedded values. At the foundation of the claim that Taylor’s theory of personal agency offers a context for nurse-person relatedness, and thus persons’ development, rest two complementary, critical notions: first, that respect for persons’ capacity to identify their own good requires that nurses attempt to understand what it is for the other to live the life he or she lives [16], and secondly, that in feeling respected by nurses, community members may engage in continuing acts of self-interpretation, leading to the identification of who they want to become, and thus, in Parse’s [13] terms, a feeling of competence.

Recall, before attempting to influence the public space, community members who experience social exclusion may benefit by consciously articulating their personal identities, as claimed by Williams, Labonte and O’Brien [23]. In specific, these investigators utilized their study, “Empowering social action through narratives of identity and culture” to shed light on “how communities strengthen their ability to take collective action on issues of their choosing, and to make positive changes in their environments”. In this study, they reported that, through storytelling, participants focused on “listening and being with the emotional content” rather than analyzing the oppressive social conditions that impact their lives. Further, the researchers concluded that, in conversing with one another about their cultural beliefs and values, participants were actively “creating themselves”, or more precisely, storytelling enabled a “conscious reconnection to and reconstitution of people’s identity”. Unfortunately, this leaves nurses with a lack of understanding about how this conscious reconnection to and reconstitution of identity was informed, or how attending to the emotional content of the stories they told one another may have made a difference to how they perceived and understood themselves.

Taylor’s [8, 21, 22, 35] theoretical contexts provide a means of understanding this personal development by depicting in more detail how an expansion of consciousness may evolve through continuing acts of interpretation about everyday experiences. More specifically, in a Taylorian sense, the Williams, Labonte and O’Brien [23] study participants may have expanded their awareness of standards embedded in their community practices, or cultures, and thus knew explicitly values that, prior to self-interpretation, had only been dimly known. More precisely, they may have articulated their particular values, hence individual identities, in a strong and original manner, as they conversed and listened to the emotional content of one another’s narratives. In a discernment process that embodies a growing awareness of values and judgment about how to act in order to express those values, the study participants may have, as Williams, Labonte and O’Brien reported, made a conscious reconnection to and reconstitution of their identities. Moreover, participants’ capacity to engage in this clarification process may have been related to feeling respected, yielding within each study participant an interior sense of competence in becoming one’s own person.

Nurses who establish relational narratives with community members who experience health inequalities may open the door for these community members to gain a discerning insight into the meaning of their experiences, as well as to evaluate whether they express their values in their everyday lives. By acting upon the advice of Williams [23], who posited that respect for others requires that one tries to understand what it is for the other to live the life he or she lives, nurses who exercise respect may encourage community members to continue in their acts of self-interpretation. Thus, nurses who engage in relational narratives may expand opportunities for persons who experience health inequalities to appraise whether they are able to live up to the standards they seek to realize in living a coherent life. Bring back to mind Taylor’s [8] assumption that in being a person, one wants to set standards for the self, as well as evaluate whether one has met those standards. In perceiving that another is trying to understand what it is to live one’s life, a person may persevere in the interpretative process, thus gaining a discerning insight into how and why life experiences may unfold as they do, whether one has met personal standards, and, where discrepancies exist, what resources are needed to realize one’s good. In knowing explicitly one’s personal identity, community members individually and collectively may persuade policy-makers to distribute public resources to mitigate harmful social conditions, which interfere with the pursuit of individual and community goals.

## IMPLICATIONS FOR NURSING EDUCATION

As the relational narrative is recognized as the epistemology of nursing [39], nurse educators must attend explicitly to the preparation of nursing students for inter-subjective encounters by which persons discern their values, and thus transform experiences of suffering into experiences of personal development. In recent decades, nurses have relied on principles, found in professional nursing codes of ethics, to guide their nursing action; however, as Vanlaere and Gastmans [40] suggested, sound nursing care can be provided only when nurses’ actions are rooted in “a moral inner attitude”, or relational approaches, such as empathy [14]. An inner attitude is enhanced through self-examination that scrutinizes the uniqueness of each experience and that results in an assignment of meaning to these experiences. The theory of virtue ethics, as presented by MacIntyre [38], embodies a framework by which student nurses may realize this assignment of meaning, owing to the theory’s premise that character traits or qualities, such as respect, are requisite to achievement of one’s purposes or goals. An aim of nursing education ought thus to be the professional development of students acquired through a support of their acquisition of moral sensitivity and a fostering of their appreciation of the “worthwhileness” [41] of their virtue-focused practices.

In a forward-thinking inquiry, Lindh, Severinsson and Berg [42] revealed that student nurses interpret moral responsibility as a relational way of being and that their relational practices are guided by an “inner compass”, comprised of “ideals, values and knowledge that translate into a striving to do good”. Moreover, the investigators’ study indicated that role models inform the student nurses’ acquisition of professional values, meaning that they identify with their clinical supervisors’ values and personal qualities. Therefore, as role models, nurse educators are given the opportunity to influence the development of professional qualities among student nurses. This character formation may enhance nursing students’ practical reasoning or prudence [35], which embodies perceptual judgments about experiences that lead to actions [43]. Even though role modelling is often a hidden component of nursing education, educators remain role models whether or not they are aware of this impact on the students [44]. In order to improve consciously the good of their practice [38], through the development of moral sensitivity among learners, educators ought to become aware of this role and engage in their own self-clarification, thus articulating their values, in addition to sharing their insights into how these values inform their educational practices [44]. Nursing has focussed on the application of ethical principles found in their codes of ethics; an integration of virtue theories into nursing education will necessitate, in addition, a willingness on the part of educators to engage in their own self-interpretation, for both their professional formation and their educational practice development.

Nurses’ engagement in relational narratives, which foster persons’ discernment of values, may be facilitated by a disposition of respect [13]. In conveying respect, nurses may encourage persons to continue acts of self-interpretation and chose actions that express their values; students in nursing thus need innovative opportunities to learn the exercise of this value. In facing the challenge of providing students with learning opportunities, nurse educators may gain support from Milton’s [45] contention that fables can be used to convey explicit yet hidden “moral maxims” that instruct members of a discipline on “what they ought to do”. Keeping in mind that fables are understood within a particular philosophical lens [45], students who examine the meaning of fables within a conceptual context of respect may gain awareness of their moral duty to regard persons’ capacity to realize their own good. In reflecting on a fable, such as the “Wind and the Sun” [46], student nurses may develop greater appreciation for the exercise of respect, for example, students may realize that persons who are confident in their being may strongly influence health policy-makers just as the sun with its warmth can more effectively persuade a person to take off her coat than can the wind with its force.

## Figures and Tables

**Table 1:** Qualitative Evidence Addressing Nurses’ Characterizations of Their Advocacy

**Authors**	Snowball (1996) [10]	Watt (1997) [11]	Vaartio, Leino-Kilpi, Salantera, Suominen (2006) [12]	Foley, Minick & Kee (2000) [17]	Chafey, Rhea & Spencer (1998) [18]
**Sample**	15 nurses in a large teaching hospital in the United Kingdom (UK). The sample included only nurses who had practiced for at least one year. The investigator believed that experience is needed to practice advocacy.	8 Australian nurses who practiced on two adult acute-care units in a large urban hospital.	22 patients and 21 nurses from four medical and four surgical units in Finland.	24 nurses who had served in a military operation in Bosnia and Hungary.	17 American nurses who practiced in hospital and community settings. Investigators’ aim was to establish a typical case of advocacy rather than to generalize.
**Purpose**	To explore nurses’ perceptions, understanding and experience of acting as a client advocate.	To explore and describe how nurses perceive the concept of client advocacy.	To describe the way advocacy is defined, the activities of advocacy, the way patients experience nursing advocacy. The focus of the interviews was procedural pain because the nursing responses are broad.	To explore the advocacy experiences of military nurses and describe their shared practices and common meanings.	To describe how nurses define and characterize advocacy and how they exercise the advocacy role.
**Method**	Data were collected by semi-structured interviews used to elicit a narrative account of participants’ perceptions, beliefs and values related to advocacy. Emergent commonalities in the data were analyzed using a qualitative approach [28].	A 50-minute semi-structured interview was conducted with each participant. Participants were prompted to describe incidents that illustrated their personal meaning of advocacy. The theory of Glasser and Strauss [31] guided data analysis.	The data were collected through 30-70 minute individual interviews, following pilot interviews. Data were analyzed inductively using qualitative content analysis as described by Miles and Huberman [30]. The analysis was conducted twice over a span of 2 months.	Data were collected by open-ended, non-structured 30-60 minute interviews. Participants were asked to narrate practices of advocacy. A constant comparative method [27] was used to analyze the data. A research team interpreted the themes and categories that emerged.	Standardized, open-ended questions explored characterizations, conditions, and values thought to be influential in advocacy during a 60 minute interview. An intuitive-analytical process [29] was used to analyze the patterns and themes that emerged.
**Findings**	All participants stressed that advocacy is “predicated” on a therapeutic relationship. Seven of the 15 participants asserted that nurses and persons > share a common humanity” [p. 72]. Two conditions were identified as fundamental to advocacy, quality nurse-client relationships and respect for inherent human characteristics, including the ability to make choices that are congruent with needs.	Two conditions were identified as fundamental to advocacy, quality nurse-client relationships and respect for inherent human qualities, including the ability to make choices that are congruent with personal needs.	Interpersonal dialogue, respect and empowerment were antecedents of nursing advocacy.	Advocacy was perceived as safeguarding client safety; engag[ing] in understand[ing] the dilemmas of the [client’s] life as [they are] actually lived”; embracing traits, such as empathy	Advocacy was described as providing clients with information to ensure they receive the care they deserve. Interpersonal relatedness was recognized as the core of advocacy, characterizing advocacy as “being a confidante”
